# Overgrazing induces alterations in the hepatic proteome of sheep (*Ovis aries*): an iTRAQ-based quantitative proteomic analysis

**DOI:** 10.1186/s12953-016-0111-z

**Published:** 2017-01-05

**Authors:** Weibo Ren, Xiangyang Hou, Yuqing Wang, Warwick Badgery, Xiliang Li, Yong Ding, Huiqin Guo, Zinian Wu, Ningning Hu, Lingqi Kong, Chun Chang, Chao Jiang, Jize Zhang

**Affiliations:** 1 Key Laboratory of Forage Grass, Ministry of Agriculture, Institute of Grassland Research, Chinese Academy of Agricultural Sciences, Hohhot, 010010 Inner Mongolia China; 2 NSW Department of Primary Industries, Orange Agricultural Institute, Orange, NSW 2800 Australia; 3 College of Life Sciences, Inner Mongolia Agricultural University, Hohhot, 010019 Inner Mongolia China

**Keywords:** Overgrazing, Liver, proteomics, Sheep

## Abstract

**Background:**

The degradation of the steppe of Inner Mongolia, due to overgrazing, has resulted in ecosystem damage as well as extensive reductions in sheep production. The growth performance of sheep is greatly reduced because of overgrazing, which triggers massive economic losses every year. The liver is an essential organ that has very important roles in multiple functions, such as nutrient metabolism, immunity and others, which are closely related to animal growth. However, to our knowledge, no detailed studies have evaluated hepatic metabolism adaption in sheep due to overgrazing. The molecular mechanisms that underlie these effects remain unclear.

**Methods:**

In the present study, our group applied isobaric tags for relative and absolute quantitation (iTRAQ)-based quantitative proteomic analysis to investigate changes in the protein profiles of sheep hepatic tissues when nutrition was reduced due to overgrazing (12.0 sheep/ha), with the goal of characterizing the molecular mechanisms of hepatic metabolism adaption in sheep in an overgrazing condition.

**Results:**

The body weight daily gain of sheep was greatly decreased due to overgrazing. Overall, 41 proteins were found to be differentially abundant in the hepatic tissue between a light grazing group and an overgrazing group. Most of the differentially expressed proteins identified are involved in protein metabolism, transcriptional and translational regulation, and immune response. In particular, the altered abundance of kynureninase (KYNU) and HAL (histidine ammonia-lyase) involved in protein metabolic function, integrated with the changes of serum levels of blood urea nitrogen (BUN) and glucose (GLU), suggest that overgrazing triggers a shift in energy resources from carbohydrates to proteins, causing poorer nitrogen utilization efficiency. Altogether, these results suggest that the reductions in animal growth induced by overgrazing are associated with liver proteomic changes, especially the proteins involved in nitrogen compounds metabolism and immunity.

**Conclusions:**

This provides new information that can be used for nutritional supplementation to improve the growth performance of sheep in an overgrazing condition.

**Electronic supplementary material:**

The online version of this article (doi:10.1186/s12953-016-0111-z) contains supplementary material, which is available to authorized users.

## Background

The Inner Mongolian steppe is the most important region for mutton and milk production in China [1]. However, this natural grassland has been severely damaged by overgrazing in recent decades [2]. The degradation of the Inner Mongolian steppe due to overgrazing also damages the ecosystem and has extensively reduced animal productions. Increasing evidence shows that the quantity and quality of herbage and growth of sheep were substantially reduced due to overgrazing [3, 4]. Increasing grazing intensity elevated the odour source of volatile organic compounds in grassland plants and altered the morphological response of the host plants [5]. A previous study demonstrated that stocking rate rather than management system determined the ecological sustainability of pastoral livestock system [6]. Most importantly, the body weight gain was significantly decreased (up to 55%) due to overgrazing during the grazing season in multiple years studies [7, 8]. However, few studies have been conducted on the effects of overgrazing on the metabolic alterations related to sheep growth due to the research gap between animal nutritional metabolism and grazing [9, 10]. Furthermore, the molecular mechanism of the growth reduction induced by overgrazing in sheep is unknown.

The liver plays a central role in the regulation of the metabolism of carbohydrates, proteins, lipids and other nutrients in animals. Additionally, the liver has multiple other physiological functions in the body including immune response, regulating inflammation and the removal of xenobiotics [11, 12]. A previous gene array study on beef cows demonstrated marked hepatic responses to high or low grazing herbage allowances of native grasslands, including genes associated with glucogenesis, fatty acid oxidation, cell growth, DNA replication and transcription [13]. The proteomic profile of hepatic tissue in goats fed a high-grain diet demonstrated that an altered expression of hepatic proteins was related to amino acids metabolism [14]. To date, most studies on grazing animal have simply focused on body weight gain relating to intake based on the quality and quantity of herbage. However, few detailed studies have been conducted on the hepatic response to overgrazing in sheep.

Studies have shown lack of correlation between mRNA and protein expression abundance due to RNA editing and posttranslational modifications [15, 16]. Thus, the elucidation of protein expression is more imperative [17]. Previous research demonstrated that changes in animal growth performance are closely related to alterations in the protein expression in the hepatic tissue [18, 19]. A number of enzymes or functional proteins in the liver participate in physiological processes relevant to immunity, detoxification, nutrient metabolism, and others [20]. It is not practical to simultaneously measure all protein expressions of hepatic tissue using classical biotechnologies such as western blot, immunohistochemical staining or ELISA.

Thus, we hypothesized that overgrazing can confer negative effects on the hepatic protein expression. Therefore, the objective of this study was to identify and characterize candidate proteins that were differentially induced in the livers of sheep from an overgrazed pasture during the grazing season using a label-based iTRAQ procedure (isobaric tags for relative and absolute quantitation) followed by LC-MS/MS.

## Results

### Effect of overgrazing on herbage and animals

The herbage crude protein (CP) and acid detergent lignin (ADL) contents in the overgrazing (OG) group were 34.4 and 19.6% greater, respectively, than those in the light grazing (LG) group over the grazing period (*P* = 0.003; *P* = 0.049). However, the gross energy and nitrogen free extract (NFE) contents were significantly decreased in the OG group than the LG group (16.42 ± 0.37 kJ/g vs. 17.53 ± 0.12 kJ/g, *P* = 0.008; 40.2 ± 1.5 g/kg vs. 46.9 ± 4.2 g/kg, *P* = 0.048). There was no significant effect of overgrazing on neutral detergent fibre (NDF) or acid detergent fibre (ADF). However, NDF tended to decrease in the OG group (*P* = 0.096). A full description of the effects of overgrazing on herbage and animal growth performance are given in Additional file 1: Table S1 and Table 1, respectively.

**Table 1:** Effect of overgrazing on the growth and immune organ indexes of sheep

	Groups		
	LG^c^	OG^d^	*P* value
Daily gain (g)	153 ± 13^a^	126 ± 9^b^	0.042
Carcass weight cold (kg)	22.8 ± 1.2^a^	20.0 ± 1.2^b^	0.047
Index of spleen (%)^c^	0.43 ± 0.02^a^	0.34 ± 0.03^b^	0.004
Index of liver (%)^c^	3.36 ± 0.11^a^	2.98 ± 0.03^b^	0.010

^a, b^ Values within a row not sharing a common superscript letter indicate significant difference at *P* < 0.05. Numbers are means ± SD. (Daily gain: *n* = 12 for LG, *n* = 48 for OG; *n* = 3 for indices of immune organs). Immune organ indexes were calculated as the ration of organ weight to body weight

^c^ *LG* light grazing

^d^ *OG* overgrazing

In this study, all sheep had similar body weights (31.5 ± 4.5 kg) at the beginning of the grazing experiment. Throughout the experimental grazing period (total 90 d), the OG sheep had a 21.4% reduction in daily weight gain (27 g) (*P* = 0.042) and 14.0% reduction in carcass weight (2.8 kg) (*P* = 0.047) (Table 1). Additionally, two most important organ indices (calculated based on the weight of the spleen and liver) in the OG group were significantly lower than those in the LG group (*P* = 0.004; *P* = 0.010) (Table 1).

### Effect of overgrazing on biochemical parameters of serum

The effects of overgrazing on the biochemical parameters of serum in sheep are shown in Table 2. The alanine aminotransferase (ALT) and aspartate aminotransferase (AST) activities in serum were significantly increased in response to overgrazing compared with light grazing (*P* = 0.038; *P* = 0.009). Furthermore, the serum concentration of blood urea nitrogen (BUN) was significantly higher in the OG group than in the LG group (*P* = 0.046). In contrast, the levels of total protein (TP), glucose (GLU), non-esterified fatty acid (NEFA) and insulin-like growth factor 1 (IGF-1) in the serum were significantly decreased in response to overgrazing compared with light grazing (*P* = 0.044; *P* = 0.017; *P* = 0.032; *P* = 0.018). However, the levels of triglyceride (TG) and cholesterol (CHOL) were similar between the two groups (*P* > 0.05).

**Table 2:** Effect of overgrazing on serum biochemical parameters of sheep

	Groups		
	LG^c^	OG^d^	*P* value
ALT (IU/L)^e^	20.73 ± 9.41^b^	45.97 ± 10.86^a^	0.038
AST (IU/L)^f^	164.93 ± 28.94^b^	386.37 ± 75.32^a^	0.009
TP (mmol/L)^g^	73.03 ± 4.05^a^	64.23 ± 3.32^b^	0.044
BUN (mmol/L)^h^	5.03 ± 0.42^b^	6.77 ± 0.96^a^	0.046
GLU (mmol/L)^i^	7.80 ± 0.72^a^	5.71 ± 0.58^b^	0.017
TG (mmol/L)^j^	0.36 ± 0.06	0.35 ± 0.07	0.835
CHOL (μmol/l)^k^	1.88 ± 0.84	1.44 ± 0.11	0.417
NEFA (mmol/L)^l^	0.61 ± 0.01^a^	0.44 ± 0.09^b^	0.032
IGF-1 (ng/mL)^m^	30.76 ± 4.01^a^	21.56 ± 4.18^b^	0.018

^a, b^ Values within a column not sharing a common superscript letter indicate significant difference at *P* < 0.05. Numbers are means ± SD. (*n* = 6)

^c^ *LG* light grazing

^d^ *OG* overgrazing

^e^ *ALT* alanine aminotransferase

^f^ *AST* aspartate aminotransferase

^g^ *TP* total protein

^h^ *BUN* blood urea nitrogen

^i^ *GLU* glucose

^j^ *TG* triglyceride

^k^ *CHOL* cholesterol

^l^ *NEFA* non-esterified fatty acid

^m^ *IGF-1* insulin-like growth factor 1

### Identification and comparison of proteins of differential abundance

Using iTRAQ analysis, a total of 27,287 peptide spectral matches were found, and 2,153 proteins were identified within the FDR (false discover rate) of 1% (Additional file 2: Table S2). Following the statistical analysis, 45 proteins were found to be differentially expressed in hepatic tissue between the LG and OG groups, with 8 being up-regulated and 37 down-regulated (Additional file 3: Table S3).

**Fig. 1:**
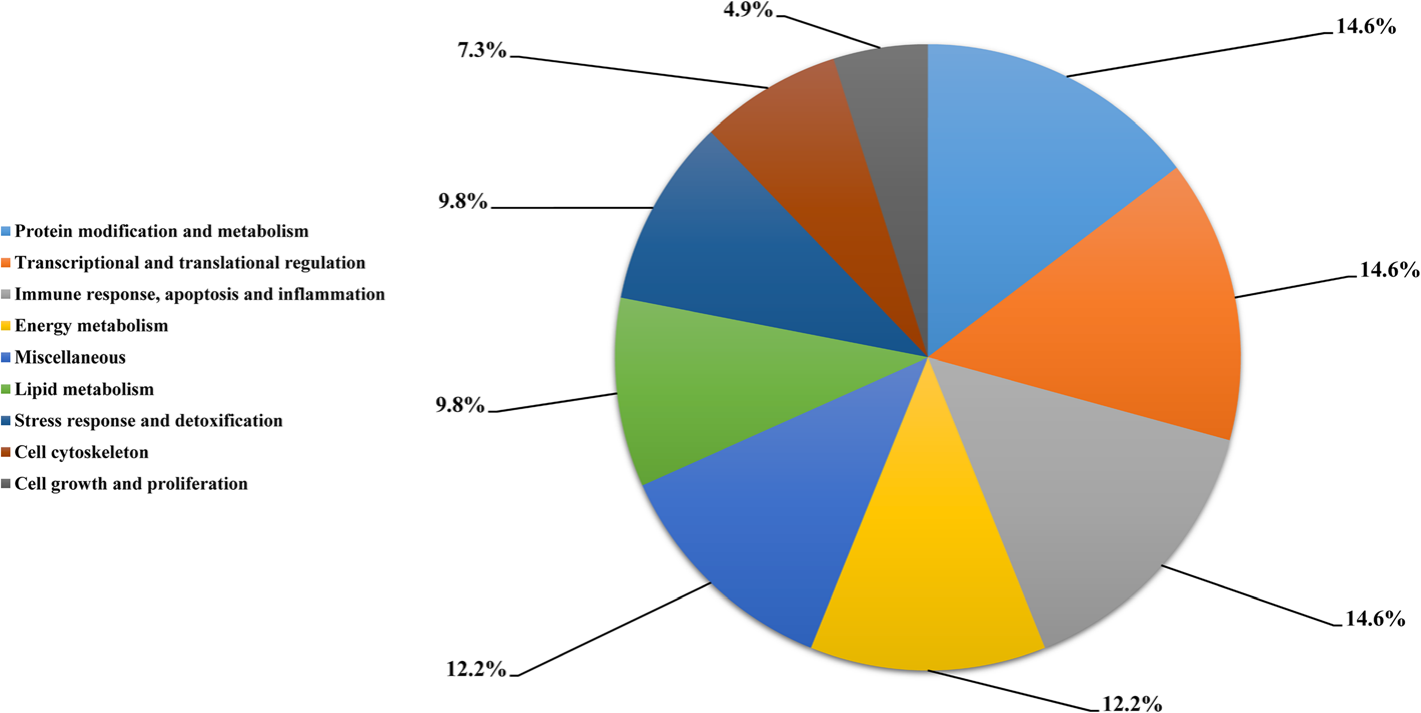
Functional classification of the proteins of differential abundance identified from the hepatic tissues of sheep

A total of 41 proteins of differential abundance were grouped into nine classes based on putative functions: protein modification and metabolism (14.6%), transcriptional and translational regulation (14.6%), immune response, apoptosis and inflammation (14.6%), energy metabolism (12.2%), miscellaneous (12.2%), lipid metabolism (9.8%), stress response and detoxification (9.8%), cell cytoskeleton (7.3%) and cell growth and proliferation (4.9%) (Fig. 1). Those related to protein modification and metabolism, transcriptional and translational regulation, immune response, apoptosis and inflammation, and energy metabolism were predominant and accounted for approximately 55% of the differentially-expressed proteins. A comparison of proteins of differential abundance with functional grouping between the two grazing intensities indicated that more protein species were down- regulated in the overgrazing sheep (34 versus 7, respectively) (Table 3). Most importantly, the protein species that participated in energy metabolism, lipid metabolism, cell cytoskeleton, and cell growth and proliferation were found to be down-regulated in OG sheep in the present study.

**Table 3:** List of differentially expressed proteins in hepatic samples from overgrazing group and light grazing group

Accession^a^	Description^b^	Gene symbol	Fold change	*p*-value	Biological process GO term
Energy metabolism					
W5NYL7	Uncharacterized protein OS = Ovis aries GN = MTHFD1L PE = 3 SV = 1 - [W5NYL7_SHEEP]	MTHFD1L	0.69	0.0403	ATP binding
Q5MIB6	Glycogen phosphorylase, brain form OS = Ovis aries GN = PYGB PE = 2 SV = 3 - [PYGB_SHEEP]	PYGB	0.82	0.0397	Carbohydrate metabolic process
W5PM30	Uncharacterized protein OS = Ovis aries GN = CREB3L3 PE = 4 SV = 1 - [W5PM30_SHEEP]	CREB3L3	0.72	0.0223	Maintaining systemic energy homeostasis
W5NUJ4	Uncharacterized protein OS = Ovis aries GN = NEK9 PE = 4 SV = 1 - [W5NUJ4_SHEEP]	NEK9	0.82	0.0185	ATP binding
B9VXW5	Mammalian target of rapamycin OS = Ovis aries PE = 2 SV = 1 - [B9VXW5_SHEEP]	mTOR	0.83	0.0051	ATP binding
Protein modification and metabolism					
W5PJN6	Kynureninase OS = Ovis aries GN = KYNU PE = 3 SV = 1 - [W5PJN6_SHEEP]	KYNU	1.21	0.0148	Tryptophan catabolic process
W5Q678	Uncharacterized protein OS = Ovis aries GN = SEC24D PE = 4 SV = 1 - [W5Q678_SHEEP]	SEC24D	0.81	0.0369	Intracellular protein transport
W5QHW3	Uncharacterized protein OS = Ovis aries GN = THNSL2 PE = 4 SV = 1 - [W5QHW3_SHEEP]	THNSL2	0.60	0.0349	Serine binding
W5PFC9	Uncharacterized protein (Fragment) OS = Ovis aries GN = LOC101117129 PE = 4 SV = 1 - [W5PFC9_SHEEP]	LOC101117129	0.78	0.0099	Enzyme inhibitor activity
W5QF53	Uncharacterized protein OS = Ovis aries GN = ZFAND2B PE = 4 SV = 1 - [W5QF53_SHEEP]	ZFAND2B	0.67	0.0095	Maintain cellular folding capacity
W5PRG9	Histidine ammonia-lyase OS = Ovis aries GN = HAL PE = 3 SV = 1 - [W5PRG9_SHEEP]	HAL	0.83	0.0062	Elimination of ammonia from the substrate
Accession^a^	Description^b^	Gene symbol	Fold change	*p*-value	Biological process GO term
Lipid metabolism					
W5PMR6	Uncharacterized protein OS = Ovis aries GN = DERL1 PE = 4 SV = 1 - [W5PMR6_SHEEP]	DERL1	0.73	0.0362	ApoB secretion
W5P8F9	Uncharacterized protein OS = Ovis aries PE = 4 SV = 1 - [W5P8F9_SHEEP]	None	0.74	0.0203	Lipid binding
W5PKK8	Uncharacterized protein OS = Ovis aries GN = ESYT1 PE = 4 SV = 1 - [W5PKK8_SHEEP]	ESYT1	0.81	0.0344	Lipid binding
W5Q6U0	Uncharacterized protein OS = Ovis aries GN = FASN PE = 4 SV = 1 - [W5Q6U0_SHEEP]	FASN	0.79	0.0147	De novo synthesis of fatty acids
Transcriptional and translational regulation					
W5P328	Uncharacterized protein OS = Ovis aries GN = EIF2A PE = 4 SV = 1 - [W5P328_SHEEP]	EIF2A	1.26	0.0487	Regulation of translation
W5P2A1	Uncharacterized protein OS = Ovis aries PE = 3 SV = 1 - [W5P2A1_SHEEP]	None	0.81	0.0482	Translational elongation
W5PLU3	Uncharacterized protein OS = Ovis aries GN = ZNF207 PE = 4 SV = 1 - [W5PLU3_SHEEP]	ZNF207	0.68	0.0389	Transcription regulation and chromatin organization
W5PHI1	Uncharacterized protein (Fragment) OS = Ovis aries GN = MRPL3 PE = 3 SV = 1 - [W5PHI1_SHEEP]	MRPL3	0.59	0.0325	Structural constituent of ribosome
W5PTA9	Uncharacterized protein OS = Ovis aries GN = DCPS PE = 4 SV = 1 - [W5PTA9_SHEEP]	DCPS	0.69	0.0039	Regulation of RNA stability
B0FZM0	Ribosomal protein L14-like protein (Fragment) OS = Ovis aries PE = 2 SV = 1 - [B0FZM0_SHEEP]	None	0.75	0.0028	Structural constituent of ribosome
Accession^a^	Description^b^	Gene symbol	Fold change	*p*-value	Biological process GO term
Immune response, apoptosis and inflammation					
W5QAE0	Uncharacterized protein OS = Ovis aries GN = TMBIM6 PE = 3 SV = 1 - [W5QAE0_SHEEP]	TMBIM6	1.31	0.0247	Intrinsic apoptotic signaling pathway
W5PKK4	Uncharacterized protein OS = Ovis aries GN = CCAR2 PE = 4 SV = 1 - [W5PKK4_SHEEP]	CCAR2	1.51	0.0402	Positive regulation of apoptotic process
W5PUV3	Uncharacterized protein OS = Ovis aries GN = NT5E PE = 3 SV = 1 - [W5PUV3_SHEEP]	NT5E	0.83	0.0387	Marker of lymphocyte differentiation
W5P8T5	Uncharacterized protein OS = Ovis aries GN = PPM1B PE = 3 SV = 1 - [W5P8T5_SHEEP]	PPM1B	0.73	0.0358	Protein serine/threonine phosphatase activity
W5NZ57	Proteasome subunit beta type OS = Ovis aries GN = PSMB10 PE = 3 SV = 1 - [W5NZ57_SHEEP]	PSMB10	0.78	0.0344	T cell proliferation
W5Q0Z8	Uncharacterized protein OS = Ovis aries GN = TRIM56 PE = 4 SV = 1 - [W5Q0Z8_SHEEP]	TRIM56	0.29	0.0011	Regulator of host innate immunity
Stress response and detoxification					
W5PGA7	UDP-glucuronosyltransferase OS = Ovis aries GN = UGT2B7 PE = 3 SV = 1 - [W5PGA7_SHEEP]	UGT2B7	0.79	0.0376	Elimination of potentially toxic xenobiotics and endogenous compounds
W5PWA2	Uncharacterized protein OS = Ovis aries PE = 3 SV = 1 - [W5PWA2_SHEEP]	None	1.24	0.0140	Oxidoreductase activity
W5PKE4	Uncharacterized protein OS = Ovis aries PE = 4 SV = 1 - [W5PKE4_SHEEP]	None	0.56	0.0024	Oxidation reduction process
W5P214	Transgelin OS = Ovis aries GN = TAGLN PE = 3 SV = 1 - [W5P214_SHEEP]	TAGLN	0.82	0.0006	Stress response related
Cell growth and proliferation					
W5Q8Q6	Uncharacterized protein OS = Ovis aries GN = KANK2 PE = 4 SV = 1 - [W5Q8Q6_SHEEP]	KANK2	0.81	0.0474	Promotion of cell proliferation
W5P0W0	Uncharacterized protein (Fragment) OS = Ovis aries GN = IST1 PE = 4 SV = 1 - [W5P0W0_SHEEP]	IST1	0.72	0.0015	Cytokinesis
Accession^a^	Description^b^	Gene symbol	Fold change	*p*-value	Biological process GO term
Cell cytoskeleton					
W5PK38	Uncharacterized protein (Fragment) OS = Ovis aries GN = VASP PE = 4 SV = 1 - [W5PK38_SHEEP]	VASP	0.76	0.0135	Actin cytoskeleton organization
W5Q5K3	Uncharacterized protein (Fragment) OS = Ovis aries GN = NCKAP1 PE = 4 SV = 1 - [W5Q5K3_SHEEP]	NCKAP1	0.69	0.0022	Regulation of actin cytoskeleton
W5Q8P7	Uncharacterized protein (Fragment) OS = Ovis aries GN = SNTB1 PE = 4 SV = 1 - [W5Q8P7_SHEEP]	SNTB1	0.59	0.0004	Structural molecule activity
Miscellaneous					
W5PEV3	Uncharacterized protein OS = Ovis aries GN = ARSD PE = 4 SV = 1 - [W5PEV3_SHEEP]	ARSD	1.24	0.0138	Disease marker
W5NRU6	Uncharacterized protein OS = Ovis aries GN = MYADM PE = 4 SV = 1 - [W5NRU6_SHEEP]	MYADM	1.36	0.0102	Disease marker
W5QGB6	Uncharacterized protein OS = Ovis aries GN = GCHFR PE = 4 SV = 1 - [W5QGB6_SHEEP]	GCHFR	0.79	0.0386	Negative regulation of biosynthetic process
W5P2J9	Uncharacterized protein OS = Ovis aries PE = 3 SV = 1 - [W5P2J9_SHEEP]	RUSC1	0.77	0.0342	Transferase activity
W5PMB1	Uncharacterized protein OS = Ovis aries GN = SNX3 PE = 4 SV = 1 - [W5PMB1_SHEEP]	SNX3	0.82	0.0197	Iron homeostasis

^a^Uniprot_Ovis aries _27110_20151123 database accession number

^b^The name of the protein exclusive of the identifier that appears in the database

### GO annotations and pathway analysis

**Fig. 2:**
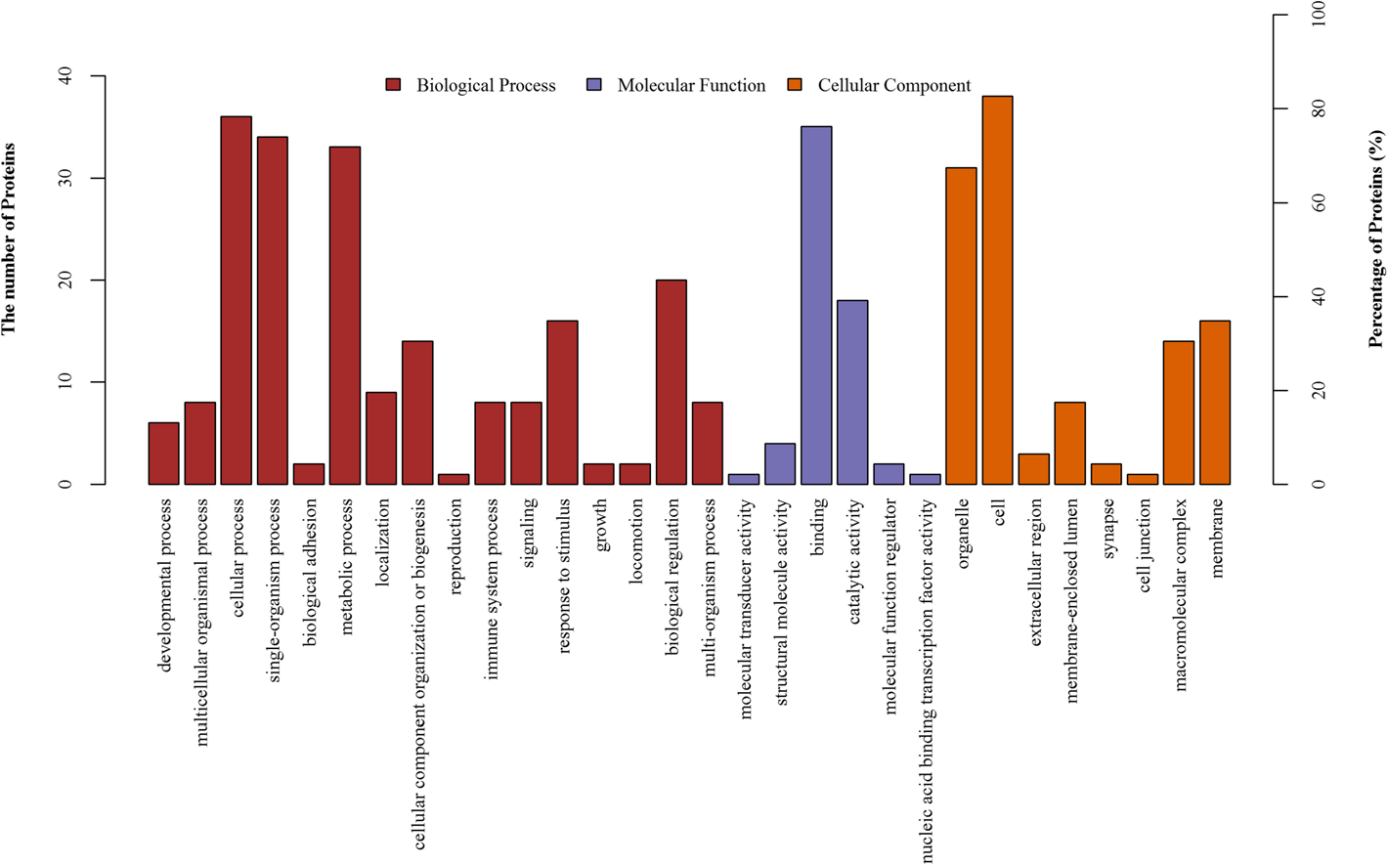
GO distribution analysis of differentially expressed proteins in hepatic tissues from OG group and LG group. OG, overgrazing; LG, light grazing. The number of proteins for each GO annotation is shown in right axis, and the proportion of proteins for each GO annotation is exhibited in left axis

In the cellular component group, the differentially expressed proteins are concentrated in the cell part and membrane-bounded organelle (Fig. 2). In the molecular functional group, the differentially expressed proteins that were binding proteins (protein binding, ion binding and heterocyclic compound binding), metabolic enzymes (hydrolase activity, transferase activity and oxidoreductase activity) and structural molecules (structural constituents of ribosomes) were ranked at the top of the category occupancy, suggesting that their related functions were important in the livers of the sheep (Fig. 2). In the biological process category, the proteins that participate in cellular processes (macromolecule metabolism), single-organism process and metabolism (nitrogen compound metabolism, heterocycle metabolism and lipid metabolism) were at the highest ratios among the differentially expressed proteins (Fig. 2), suggesting that overgrazing primarily results in changes to nutrient metabolism.

Furthermore, GO annotation and a KEGG pathway enrichment analysis were performed to determine the overrepresented biological events and to provide a primary overview of the hepatic proteome impacted by overgrazing. The DAVID 6.7 software identified highly overrepresented GO classes including cellular components, molecular functions and biological processes (Additional file 4: Table S4). For these identified differentially expressed proteins, the cellular component classifications were enriched in the cytoplasmic membrane-bounded vesicle, membrane-bounded vesicle, cytoplasmic vesicle and vesicle. According to the molecular function classifications, the differentially expressed proteins were enriched in vitamin binding, cofactor binding, vitamin B6 binding and pyridoxal phosphate binding. Enriched biological process classifications of the differentially expressed proteins included the heterocycle catabolic process, response to unfolded proteins, the cellular amino acid catabolic process and response to protein stimulus, which implies that the protein metabolic process was greatly influenced in the livers of sheep in an overgrazing condition. A KEGG analysis showed that insulin signaling was significantly enriched (*P* = 0.047) in the identified pathways, which indicates that overgrazing had affected glucose and energy metabolism in the sheep.

### Target proteomics validation of important proteins of differential abundance

**Fig. 3:**
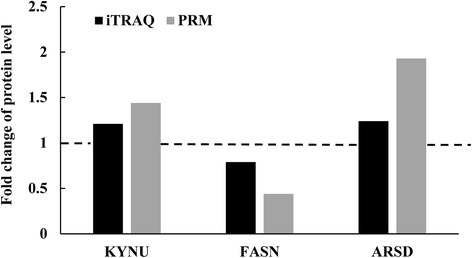
Expression patterns of selected protein candidates in the hepatic tissue of OG (overgrazing) group compared with LG (light grazing) group using iTRAQ analysis and PRM validation. Fold change of protein levels (the mean value of OG group/the mean value of LG group) of KYNU (kynureninase), FASN (fatty acid synthase) and ARSD (arylsulfatase D) from iTRAQ analysis and PRM validation

Three differentially expressed proteins (KYNU involved in amino acid catabolism, FASN involved in fatty acids synthesis, and ARSD as disease marker) were selected for validation of proteomic data at the protein level using parallel reaction monitoring (PRM). The results of PRM analysis indicated that candidate proteins show similar trends as the iTRAQ results, which implied the credibility of the proteomics data (Fig. 3).

### Effect of overgrazing on immune response and inflammatory indexes of serum

As shown in the list of differentially expressed protein species involved, those related to immune response and inflammation were ranked at one of the top putative protein functions. To further evaluate the effect of overgrazing on the immune response and inflammatory parameters of sheep, we measured the levels of primary immunoglobulins (Ig) and inflammatory cytokines in serum (Table 4). The levels of primary inflammatory cytokines, including interleukin-1β (IL-1β), interleukin-4 (IL-4) and interleukin-6 (IL-6), were greatly increased in the OG group compared with the LG group (*P* = 0.004; *P* = 0.007; *P* = 0.001). While, the levels of IgA, IgG and interferon-γ (IFN-γ) were significantly lower in the OG group than in the LG group (*P* = 0.050; *P* = 0.022; *P* = 0.036).

**Table 4:** Effect of overgrazing on the immune responses and inflammatory cytokines of sheep

	Groups		
	LG^c^	OG^d^	*P* value
IL-1β (pg/mL)^e^	10.34 ± 0.72^b^	22.90 ± 3.68^a^	0.004
IL-4 (pg/mL)^f^	21.38 ± 2.19^b^	29.06 ± 1.43^a^	0.007
IL-6 (pg/mL)^g^	90.61 ± 22.03^b^	222.50 ± 10.81^a^	0.001
IFN-γ (pg/mL)^h^	30.76 ± 4.01^a^	21.56 ± 4.18^b^	0.050
IgA (g/L)^i^	0.73 ± 0.06^a^	0.54 ± 0.06^b^	0.022
IgG (g/L)^j^	19.85 ± 1.95^a^	15.19 ± 1.73^b^	0.036

^a, b^ Values within a column not sharing a common superscript letter indicate significant difference at *P* < 0.05. Numbers are means ± SD. (*n* = 6)

^c^ *LG* light grazing

^d^ *OG* overgrazing

^e^ *IL-1β* interleukin-1β

^f^ *IL-4* interleukin-4

^g^ *IL-6* interleukin-6

^h^ *IFN-γ* interferon-γ

^i^ *IgA* immunoglobulin A

^j^ *IgG* immunoglobulin G

## Discussion

The liver is a vital organ that plays important roles in multiple physiological functions such as nutrient metabolism, detoxification, immune response and others. Any change in dietary components can be closely related to animal growth, which is reflected in variations of hepatic protein expressions in most cases. Whether overgrazing has a direct effect on the functional hepatocytes (thereby causing synthetic and metabolic changes in the sheep’s liver) remains unclear. Substantially less is understood regarding the molecular mechanism of hepatic response in sheep with reduced nutrition due to overgrazing. In the present study, an iTRAQ-based quantitative proteomic analysis integrated with biochemical and immune analyses was applied to investigate the hepatic response in overgrazed sheep. When integrated with the proteomic profiles, biochemical and immune detections, these data suggest that overgrazing triggers a shift in energy resources from carbohydrates to proteins, which results in the impairment of nutrient metabolism (protein and lipid) and immunity, which may be the reasons for reduced growth in sheep.

Serum levels of ALT and AST serve as important indicators of hepatic health status and were found to be greatly elevated in OG sheep, indicating that overgrazing had a severely negative effect on hepatic function [21]. Additionally, a higher level of BUN in the serum of the OG group suggested that overgrazing was associated with poorer nitrogen utilization efficiency [22]. Furthermore, a decreased serum level of TP and an increased serum level of GLU in OG sheep during the period may have been caused by a shift in energy resources from carbohydrates to proteins due to the imbalance in protein and fermentable carbohydrates in the herbage [20]. Moreover, lower levels of NEFA and IGF-1 were observed in the OG group, which indicated that fatty acid biosynthesis and anabolic effects in sheep were reduced due to overgrazing [20]. Both above nutrients are very closely related to animal growth.

The liver is an essential nutrient metabolism organ in which, nitrogen compounds metabolism takes a central role. Nitrogen compounds metabolism, including protein metabolism, amino acid metabolism, ammonia toxic elimination and others, can thus be an important indicator of hepatocyte health. [23]. In this study, nearly all six differentially expressed protein species related to protein modification and metabolism were decreased in OG sheep with the exception of KYNU (kynureninase). Among these proteins, KYNU is an enzyme within the tryptophan metabolism pathway [24]. A previous study demonstrated that tryptophan was the third most limiting amino acid in growing lambs, and its inadequate supply or increased catabolism *in vivo* can trigger limited protein deposition and the elevation of urinary N excretion [25]. The up-regulation of KYNU was observed in the livers of OG sheep and is consistent with the higher serum level of BUN in the present study. HAL (histidine ammonia-lyase) catalyzes the elimination of ammonia from the substrate to form (*E*)-urocanate [26]. The down-regulation of this protein indicates that overgrazing may result in perturbations in ammonia detoxification in hepatocytes, which can be another reason for the increased concentration of BUN in the OG group. Taken together, the expression changes of KYNU and HAL integrated with the results of serum levels of BUN and GLU indicate that overgrazing triggers a shift in energy resources from carbohydrates to proteins causing poorer nitrogen utilization efficiency. A GO annotation enrichment analysis done in the present study also showed that cellular amino acid catabolic process is overrepresented in the hepatic proteome of sheep due to overgrazing. The down-regulation of the other four proteins involved in protein metabolism, including SEC24D (SEC24 homologue D, COPII coat complex component), THNSL2 (threonine Synthase-Like 2), LOC101117129 (UniProt database accession W5PFC9) and ZFAND2B (Zinc finger, AN1-Type Domain 2B), may reflect reduced protein transport, binding and folding capacity [27–29].

Energy production is one of the key functions of the liver, which participates in a number of physiological processes. However, energy production and mitochondrial function are usually found to be impaired in hepatic dysfunction [30]. PYGB (glycogen phosphorylase, brain form) serves as a glucose metabolism protein and contributes to the regulation of carbohydrate metabolism [31]. CREB3L3 (cAMP-responsive element-binding protein 3-like 3) maintains systemic energy homeostasis throughout the entire body [32]. mTOR (mammalian target of rapamycin) is a central signaling molecule that impacts most cellular functions including energy metabolism promotion [33]. MTHFD1L (methylenetetrahydrofolate dehydrogenase (NADP+ dependent) 1-like) and NEK9 (NIMA-related kinase 9) are ATP binding proteins that are involved in mitochondrial function and DNA replication, respectively [34, 35]. These proteins were both down-regulated in OG sheep, suggesting that overgrazing interferes with energy production and metabolism; this is consistent with the decreased serum GLU level in OG sheep in the present study.

The down-regulated proteins DERL1, a lipid binding protein (UniProt database accession W5P8F9), ESYT1 (extended synaptotagmin-like protein 1) and FASN (fatty acid synthase) are classified as lipid metabolism proteins based on their primary function. Of these proteins, DERL1 is a putative dislocon component in the ER (endoplasmic reticulum) membrane that plays an important role in ApoB secretion [36]; FASN stimulates the de novo synthesis of fatty acids [37]. The down-regulation of these proteins implies decreased lipid metabolism in the OG group and is consistent with the finding in this study of a lower serum level of NEFA observed in OG sheep.

The liver is an important immune organ in the body that plays indispensable roles in immune response, apoptosis and inflammatory reactions [38]. In this study, two protein species related to apoptosis were up-regulated in the OG group. TMBIM6 (transmembrane BAX inhibitor motif containing 6) promotes apoptosis in prolonged stress or severe conditions [39]; CCAR2 (cell cycle and apoptosis regulator 2) leads to increased level of p53-mediated apoptosis and DNA damage [40]. The up-regulation of TMBIM6 and CCAR2 indicates that overgrazing may lead to a long-term stressful situation and trigger apoptosis in sheep hepatocytes. Other proteins involved in the immune response, including NT5E (5’-nucleotidase, ecto), PPM1B (protein phosphatase, Mg^2+^/Mn^2+^ dependent, 1B), PSMB10 (proteasome subunit beta 1) and TRIM56 (tripartite motif containing 56), are down-regulated, which suggests that the sheep immunity is suppressed during overgrazing, thus increasing the likelihood of bacterial or viral infection and reduced growth performance [41, 42]. These observations were consistent with the reduced immune organ indexes and serum levels of immunoglobulins and the increased concentrations of inflammatory cytokines in OG sheep. Moreover, the reduced abundance of proteins relevant to stress response and detoxification, including UGT2B7 (UDP-glucuronosyltransferase), an oxidoreductase (UniProt database accession W5PKE4), and TAGLN (transgelin), were observed in the OG group, which indicates that long-term overgrazing may lead to oxidative stress and that it inhibits detoxification in the sheep liver [43, 44].

Cytoskeletal proteins play a crucial role in liver protection and maintaining both the cellular structure and integrity of hepatocytes [45]. In this study, three differential protein species related to the cytoskeleton were down-regulated in the livers of OG sheep. VASP (vasodilator-stimulated phosphoprotein) is associated with filamentous actin formation and plays a widespread role in cell adhesion and motility [46]. NCKAP1 (NCK-associated protein 1) is an integral membrane protein that regulates actin cytoskeleton organization [47]. SNTB1 (beta-1-syntrophin) is a peripheral membrane protein that is associated with mediating high-density lipoprotein (HDL) metabolism in the liver [48]. This is consistent with the elevated AST and ALT serum levels in the OG group of this study, which may interfere with liver protection and normal hepatocytes structure. Other proteins relevant to cell growth and proliferation, including KANK2 (KN motif and ankyrin repeat domain 2) and IST1 (increased sodium tolerance 1), are also down-regulated and may harm the regeneration of hepatocytes in OG sheep due to overgrazing [49, 50]. Furthermore, the reduced abundance of proteins involved in transcriptional and translational regulation, including a ribosomal protein (UniProt database accession W5P2A1), ZNF207 (zinc finger protein 207), MRPL3 (mitochondrial ribosomal protein L3), DCPS (decapping enzyme, scavenger) and ribosomal protein L14-like protein, was observed in the OG group, which indicates a decreased capacity for protein metabolism to provide sufficient nutritional nitrogen compounds for growth [51–53].

## Conclusions

In summary, the present proteomic analysis demonstrated that overgrazing leads to differential abundances of a number of hepatic proteins in sheep. The functional groupings of those altered proteins are primarily related to protein metabolism, transcriptional and translational regulation, and immune response. Some of the other proteins are involved in nutrient metabolism (energy and lipid metabolism), stress response, and cellular functions (cell cytoskeleton, cell growth and proliferation). Additionally, biochemical and immune analyses provided sufficient physiological evidence. All results obtained from the present study suggest that overgrazing induces a shift in energy resources from carbohydrates to proteins, causing the impairment of nutrient metabolism (protein and lipid) and immunity, which may be the reasons for the reduced growth in sheep. Future studies will investigate the application of nutritional supplementation to improve the growth performance of sheep in overgrazing conditions.

## Methods

### Study area and experimental design

This study was conducted by the Institute of Grassland Research, Chinese Academy of Agricultural Science, Hohhot in the Xilin River Basin, Inner Mongolia Autonomous Region, China (116°32’ E, 44°15’ N). There was 8 ha of an experimental site with pasture dominated by three grass species: *Leymus chinensis*, *Stipa krylovii* and S. grandis.

A total of 60 Uzhumchin wethers were used in the present study. The sheep were born in summer 2013 and at the initiation of the grazing experiment in June were approximately 24 months old with an average live weight of 31.5 ± 4.5 kg. Water and minerals in lick stones were provided *ad libitum* during the grazing experiment.

The duration of the grazing experiment was 90 days from June 10^th^ 2015 to September 5^th^ 2015. There were a total of 6 plots (1.33 ha per plot) in the experimental site (3 plots per each grazing intensity), comprising a light grazing group (4 sheep per plot) and an overgrazing group (16 sheep per plot). Therefore, two different grazing intensities were realized: 3.0 (light grazing, LG) and 12.0 sheep/ha (overgrazing, OG).

Herbage samples were obtained monthly and combined at the end of the grazing experiment for chemical composition analysis. The herbage sample collection method was followed a previous study [6]. All details are described in Additional file 5. The content of CP, gross energy, NFE, NDF, ADF and ADL were determined in the herbage sample following the protocol of a previous study [7].

### Data and sample collection

The live weight of all animals was measured at day 0 and day 90 (end of experiment, after 12 h of fasting) of the grazing season, and the mean daily gain was calculated. At the termination of the experiment, 2 sheep per plot in both groups (*n* = 6) were randomly chosen for blood collection. Each blood sample was collected from the cervical vein using a sterilized syringe. Sera samples were obtained via centrifugation at 2000 g for 30 min at 4 °C, then at 400 g for 10 min at 4 °C, and all sera samples were stored at −80 °C for further analysis. After blood collection, one sheep per plot (*n* = 3) was slaughtered using standard procedures established by the Chinese Academy of Agricultural Sciences. To calculate the indices of immune organs, the spleen and liver were excised and weighted, respectively. Immune organ indices were calculated as the ratio of organ weight to body weight. Hepatic samples (~2 g) were then washed with ice cold sterilized PBS, frozen in liquid nitrogen, and stored at −80 °C for further proteomic analysis.

### Biochemical, immune response and inflammation analyses

For biochemical, immune response and inflammatory parameters of the serum, the concentration of ALT, AST, TP, BUN, GLU, TG, CHOL, NEFA, IgA and IgG were measured using a fully automatic biochemistry analyser (Hitachi 7020, Tokyo, Japan); IL-1β, IL-4, IL-6, IGF-1 and IFN-γ were determined using a corresponding diagnostic kit (Nanjing Jiancheng Bioengineering Institute, Nanjing, China) according to the instructions of the manufacturer.

### Hepatic sample preparation and protein extraction

A total of six hepatic samples (one sheep per plot, three biological replications per group) were collected for protein extraction and subsequent proteomic analysis. Each hepatic sample (~0.5 g) was ground after being frozen in liquid nitrogen in a Dounce glass grinder. The grinded powder was precipitated with 10% trichloroacetic acid (TCA) (w/v) and 90% ice-cold acetone at −20 °C for 2 h. The precipitate in the sample was obtained via centrifugation at 20000 g for 30 min at 4 °C and subsequently washed with ice-cold acetone. The precipitate was then lysed in the lysis buffer [8 M urea, 30 mM 4-(2-hydroxyethyl)-1-piperazineethanesulfonic acid (HEPES), 1 mM phenylmethanesulfonyl fluoride (PMSF), 2 mM ethylene diamine tetraacetic acid (EDTA), and 10 mM dithiothreitol (DTT)]. The crude tissue extracts were centrifuged to remove the remaining debris. The tissue lysates were reduced for 1 h at 37 °C in a water bath through the addition of 10 mM DTT and then alkylated with for 1 h with the addition of 55 mM iodoacetamide in the dark. Afterwards, the lysates were precipitated by adding 4 volumes of pre-chilled acetone. The pellets were then washed three times with pre-chilled pure acetone and resuspended in the buffer (50% TEAB and 0.1% SDS). The centrifugation was repeated to remove the undissolved pellets. Subsequently, protein quantitation was performed using a Bio-Rad Bradford Protein Assay Kit (Hercules, CA, USA).

### iTRAQ experiments

Each sample was digested with modified sequence grade trypsin (Promega Corporation, Madison, WI) at a 1:30 ratio (3.3 μg trypsin: 100 μg target) overnight at 37 °C. Each isobaric tag (114, 115, 116, 117, 118 and 119) (AB Sciex, Framingham, MA, USA) was solubilized in 70 μL isopropanol and then added to its respective sample (three biological replications per group). Incubation continued for 2 h at room temperature. All labelled peptides were pooled together and separated using SCX chromatography. The eluted peptides were dried under a vacuum and analyzed via LC-MS/MS based on Q-Exactive (Thermo Scientific). All detailed procedures are described in Additional file 5.

### MS data processing and bioinformatics analysis

Peptide and protein identifications were analyzed using Proteome Discover 1.4 (Thermo Fisher Scientific) and searched with the Mascot search engine (Matrix Science, London, U.K.; version 2.3.0) against the database Uniprot_Ovis aries _27110_20151123.fasta (Nov 23rd, 2015 with 27,110 protein sequences) with the following parameters: enzyme: trypsin; variable modifications: oxidation (M), gln-pyro-glu (N-term Q), and iTRAQ8plex (Y); fixed modification: carbamidomethyl (C), iTRAQ8plex (N-term), and iTRAQ8plex (K); peptide mass tolerance: ± 20 ppm; fragment mass tolerance: 0.1 Da; maximum missed cleavages: 2. Identified peptides had an ion score above the threshold of peptide identity in Mascot, and protein identifications were accepted as the false discovery rate (FDR) ≤ 0.01 in which at least one such unique peptide match was specific for the protein. Median ratio normalization was performed to obtain the quantitative protein ratios. Proteins with a 1.2-fold change or greater (*P*-values < 0.05) were considered significant.

The Gene Ontology (GO) distribution for all proteins that were differentially expressed in the hepatic tissue of overgrazed sheep was classified using Blast2GO software (http://www.blast2go.com/). The Database for Annotation, Visualization and Integrated Discovery (DAVID) 6.7 (http://david.abcc.ncifcrf.gov/) and the Kyoto encyclopedia of genes and genomes (KEGG) data base (http://www.genome.jp/kegg/), were used to classify differentially altered proteins in significantly overrepresented pathways and GO terms.

### Target analysis by PRM

Three representing proteins of interest were selected to perform the targeted quantification and verification among all proteins of differential abundance under LG and OG conditions from sheep livers. Samples were analyzed using PRM (Additional file 6: Table S5 for settings), which is applied for proteomic data verification in a number of studies [54, 55]. Details of this method are provided in the online Additional file 5.

### Statistical analysis

Statistical analyses were performed with SPSS Statistics 17.0 (SPSS, Inc., Chicago, IL, USA). All results are expressed as the means ± SD. A group difference was analyzed using Student’s *t* test, and *P* < 0.05 was considered significant.

## Additional files

Additional file 1: Table S1. Effect of overgrazing on primary nutritional indexes of herbage. (DOCX 14 kb)
Additional file 2: Table S2. List of all proteins (*n* = 2153) identified in the study. (XLSX 148 kb)
Additional file 3: Table S3. List of all differentially expressed proteins (*n* = 45) identified in the study. (XLSX 11 kb)
Additional file 4: Table S4. Enrichment analysis of differentially expressed proteins in the hepatic tissue of sheep. (DOCX 15 kb)
Additional file 5: The detailed description of the experiment methods, including herbage sample collection, SCX chromatography, mass spectrometry, targeted protein quantitation. (DOCX 15 kb)
Additional file 6: Table S5. Parallel reaction monitoring conditions for targeted natural abundance peptides and isotopically labeled peptide standards. (DOCX 14 kb)

## Abbreviations

ADF: Acid detergent fibre
ADL: Acid detergent lignin
ALT: Alanine aminotransferase
AST: Aspartate aminotransferase
BUN: Blood urea nitrogen
CCAR2: Cell cycle and apoptosis regulator 2
CHOL: Cholesterol
CP: Crude protein
CREB3L3: cAMP-responsive element-binding protein 3-like 3
ESYT1: Extended synaptotagmin-like protein 1
FASN: Fatty acid synthase
GLU: Glucose
HAL: Histidine ammonia-lyase
IGF-1: Insulin-like growth factor 1
iTRAQ: Isobaric tags for relative and absolute quantitation
KYNU: Kynureninase
mTOR: Mammalian target of rapamycin
NDF: Neutral detergent fibre
NEFA: Non-esterified fatty acid
NEK9: NIMA-related kinase 9
NFE: Nitrogen free extract
PRM: Parallel reaction monitoring
PYGB: Glycogen phosphorylase
TG: Triglyceride
TMBIM6: Transmembrane BAX inhibitor motif containing 6
TP: Total protein
