# Supplementary material for: Overgrazing induces alterations in the hepatic proteome of sheep (Ovis aries): an iTRAQ-based quantitative proteomic analysis
Source: Proteome Sci. 2017 Jan 5;15:2. doi: 10.1186/s12953-016-0111-z (PMC5267464; doi:10.1186/s12953-016-0111-z)
Supplement: Additional file 1: Table S1. — Effect of overgrazing on primary nutritional indexes of herbage. (DOCX 14 kb) [file 12953_2016_111_MOESM1_ESM.docx]

**Tables S1 Effect of overgrazing on primary nutritional indexes of herbage**

|  | Groups | |  | |  |
| --- | --- | --- | --- | --- | --- |
|  | LG | OG | |  | *P* value |
| CP (g/kg DM) | 88.6 ± 5.4^b^ | 119.1 ± 5.7^a^ | |  | 0.003 |
| Gross energy (kJ/g DM) | 17.53 ± 0.12^a^ | 16.42 ± 0.37^b^ | |  | 0.008 |
| NFE (g/kg DM) | 46.9 ± 4.2^a^ | 40.2 ± 1.5^b^ | |  | 0.048 |
| NDF (g/kg DM) | 618.3 ± 25.9 | 530.4 ± 65.1 | |  | 0.096 |
| ADF (g/kg DM) | 290.5 ± 20.0 | 322.0 ± 13.0 | |  | 0.175 |
| ADL (g/kg DM) | 250.9 ± 27.8^b^ | 300.1 ± 13.6^a^ | |  | 0.049 |

LG = light grazing; OG = overgrazing; CP = crude protein; NFE = nitrogen free extract; NDF = neutral detergent fibre; ADF = acid detergent fibre; ADL = acid detergent lignin; Values within a column not sharing a common superscript letter indicate significant difference at *P* < 0.05. Numbers are means ± SD. (n = 3).
