# Supplementary material for: Overgrazing induces alterations in the hepatic proteome of sheep (Ovis aries): an iTRAQ-based quantitative proteomic analysis
Source: Proteome Sci. 2017 Jan 5;15:2. doi: 10.1186/s12953-016-0111-z (PMC5267464; doi:10.1186/s12953-016-0111-z)
Supplement: Additional file 4: Table S4. — Enrichment analysis of differentially expressed proteins in the hepatic tissue of sheep. (DOCX 15 kb) [file 12953_2016_111_MOESM4_ESM.docx]

**Table S4 Enrichment analysis of differentially expressed proteins in the hepatic tissue of sheep**

| Category | Term | Genes | Count | *P* value |
| --- | --- | --- | --- | --- |
| Cellular component | cytoplasmic membrane-bounded vesicle | IST1, FASN, SEC24D, GCHFR | 4 | 0.0085 |
| Cellular component | membrane-bounded vesicle | IST1, FASN, SEC24D, GCHFR | 4 | 0.0091 |
| Cellular component | cytoplasmic vesicle | IST1, FASN, SEC24D, GCHFR | 4 | 0.0150 |
| Cellular component | vesicle | IST1, FASN, SEC24D, GCHFR | 4 | 0.0160 |
| Biological process | heterocycle catabolic process | NT5E, HAL, KYNU | 3 | 0.0024 |
| Biological process | response to unfolded protein | DERL1, CREB3L3 | 2 | 0.0580 |
| Biological process | cellular amino acid catabolic process | KYNU, HAL | 2 | 0.0910 |
| Biological process | response to protein stimulus | DERL1, CREB3L3 | 2 | 0.0960 |
| Molecular function | vitamin binding | KYNU, FASN, PYGB | 3 | 0.0150 |
| Molecular function | cofactor binding | KYNU, FASN, PYGB | 3 | 0.0530 |
| Molecular function | vitamin B6 binding | KYNU, PYGB | 2 | 0.0930 |
| Molecular function | pyridoxal phosphate binding | KYNU, PYGB | 2 | 0.0930 |
| KEGG pathway | Insulin signaling pathway | FASN, MTOR, PYGB | 3 | 0.0470 |

The GO enrichment analysis were performed on the ontology of cellular component, biological process and molecular function. DAVID as selected as the tool and the adjusted *P*-value less than 0.1 was chosen as cut-off criterion suggested by the software. IST1 = increased sodium tolerance 1 homolog; FASN = fatty acid synthase; SEC24D = SEC24 family member D; GCHFR = GTP cyclohydrolase I feedback regulator; NT5E = 5'-nucleotidase ecto; HAL = histidine ammonia-lyase; KYNU = kynureninase; DERL1 = derlin 1; CREB3L3 = CAMP responsive element binding protein 3-Like 3; PYGB = glycogen phosphorylase; MTOR = mechanistic target of rapamycin.
