# Supplementary material for: Overgrazing induces alterations in the hepatic proteome of sheep (Ovis aries): an iTRAQ-based quantitative proteomic analysis
Source: Proteome Sci. 2017 Jan 5;15:2. doi: 10.1186/s12953-016-0111-z (PMC5267464; doi:10.1186/s12953-016-0111-z)
Supplement: Additional file 5: — The detailed description of the experiment methods, including herbage sample collection, SCX chromatography, mass spectrometry, targeted protein quantitation. (DOCX 15 kb) [file 12953_2016_111_MOESM5_ESM.docx]

**The detailed description of experiment methods**

**Herbage sample collection**

Herbage mass (HM) was determined and samples were taken for chemical analysis on July1^st^, August 1^st^ and September 1^st^ respectively. In each paddock one pooled sample was obtained from 3 sub-samples, taken from 1 m^2^ (1×1 m) sized transects. Herbage samples included the standing biomass cut to 1 cm stubble height, assuming to be the minimum bite height of a sheep under restricted but not starving conditions. Litter (dead material spread on the ground) was not included into the herbage sample. Pooled herbage samples were dried in a 60 °C oven for 24 h and ground through a 1 mm screen.

**SCX chromatography**

SCX chromatography was performed with following procedures. Briefly, total amount of 600 μg hepatic sample was loaded onto a strong cation exchange column (Phenomenex Luna SCX 100A) equilibrated with buffer A (10 mM KH_2_PO_4_ in 25% acetonitrile, pH 3.0) using an Agilent 1100 (Santa Clara, CA) system. The peptides were separated using a linear gradient of buffer B (10 mM KH2PO4 and 2 M KCl in 25% acetonitrile, pH 3.0) increasing to 5% after 41 min, 50% after 66 min and 100% after 71 min, at a flow rate of 1 ml/min. Elution was monitored by measuring the absorbance at 214 nm. Total of 10 fractions were collected from the eluted peptides, and each fraction was desalted with a Strata X C18 column (Phenomenex) and vacuum-dried.

**Mass Spectrometry**

Each fraction was redissloved in buffer A (2% acetonitrile, 0.1% formic acid) and centrifuged at 20,000 × g for 10 min. The final concentration of peptides in each fraction was approximately 0.25 μg/μl on average. Twenty microliter of supernatant was loaded onto an UltiMate® 3000 Nano LC system (Bannockburn, IL) by the auto sampler onto a C18 trap column (length 2 cm, inner diameter 200 μm). Peptides were eluted onto a resolving analytical C18 column (length 10 cm, inner diameter 75 μM, 5-μm particles, 300 Å) packed in-house. Samples were loaded at 15 μl/min for 4 min and eluted with a 45-min gradient at 400 nl/min from 5 to 60% buffer B (98% acetonitrile, 0.1% formic acid), separated with a 3- min linear gradient to 80% B, maintained at 80% B for 7 min, and finally returned to 5% B over 3 min. The peptides were subjected to nanoelectrospray ionization followed by tandem mass spectrometry (Q-Exactive, Thermo) coupled online to the nanoLC. Intact peptides were detected in the Orbitrap at a resolution of 70,000 full with width at half maximum (FWHM). Peptides were selected for MS/MS using high-energy collision dissociation (HCD) operating mode with a normalized collision energy setting of 28%; ion fragments were detected in the Orbitrap at 17,500 FWHM resolution. A data-dependent acquisition mode that alternated between a MS scan followed by MS/MS scans was applied for the 10 most abundant precursor ions (2^+^ to 4^+^) above a threshold ion count of 20,000 in the MS survey scan with a following Dynamic Exclusion duration of 15 s (isolation window of m/z 2.0 and a maximum ion injection time of 100 ms). The electrospray voltage applied was 1.8 kV. Automatic gain control (AGC) was used to optimize the spectra generated by the Orbitrap. The AGC target for full MS was 3E6 and 1E5 for MS2. For MS scans, the m/z scan range was 350 to 2000. For MS2 scans, the m/z scan range was 100-1800.

**Targeted protein quantitation**

Peptides from each hepatic sample were obtained as described previously in Material and Method section in the present study. A specific sequence of peptides with stable isotope marker was spiked in as the standard reference (Supplementary Table S1). PRM analysis was performed by Q-Exactive Plus mass spectrometer (Thermo Fisher Scientific, San Jose, CA, USA) with the same LC gradient setting as described in Material and Method section. The MS acquisition mode was a combination of two scan events: a full scan and a time-scheduled scan. The full scan was taken at a resolution of 70000 at m/z 200 with a scan mass range of 350 to 900 m/z, a target (AGC) of 3e6 and maximum injection fill time is 250 ms. The scheduled scan was employed at a resolution of 35000 at m/z 200, a target AGC of 3e6, and maximum injection fill time is 200 ms. Precursor ions were fragmented with normalized collision energy of 27%. All fragment ions were quantified in the orbitrap. The sum of the top three fragment ion intensities was calculated in Skyline (MacCoss Lab Software version 3.5.0) and used to estimate peptide signal intensity. Peptide concentration was calculated based on the ratio to the heavy peptide standards that were added in known quantity.
