# Supplementary material for: Overgrazing induces alterations in the hepatic proteome of sheep (Ovis aries): an iTRAQ-based quantitative proteomic analysis
Source: Proteome Sci. 2017 Jan 5;15:2. doi: 10.1186/s12953-016-0111-z (PMC5267464; doi:10.1186/s12953-016-0111-z)
Supplement: Additional file 6: Table S5. — Parallel reaction monitoring conditions for targeted natural abundance peptides and isotopically labeled peptide standards. (DOCX 14 kb) [file 12953_2016_111_MOESM6_ESM.docx]

**Tables S5 Parallel reaction monitoring conditions for targeted natural abundance peptides and isotopically labeled peptide standards.**

| Protein | Gene symbol | Peptide target sequence | Charge | m/z |
| --- | --- | --- | --- | --- |
| W5PJN6 | KYNU | YLNTGAGGLAGAFVHEK,  TVVNIITPSYIEER | 2 | 852.9388  817.4434 |
| W5Q6U0 | FASN | FDASFFGVHPK,  GPSGSQQDLVK | 2 | 626.3115  558.2882 |
| W5PEV3 | ARSD | QGYTTGLIGK,  SLLPLLR | 2 | 519.2849 |
| Isotopically-labeled standard peptide as reference | None | DSPSAPVNVT**V**R | 2 | 624.3348 |

“**V**” was isotopically labeled. KYNY = kynureninase; FASN = fatty acid synthase; ARSD = arylsulfatase D.
